# The emerging role of heart‐on‐a‐chip systems in delineating mechanisms of SARS‐CoV‐2‐induced cardiac dysfunction

**DOI:** 10.1002/btm2.10581

**Published:** 2023-08-08

**Authors:** Rick Xing Ze Lu, Yimu Zhao, Milica Radisic

**Affiliations:** ^1^ Institute of Biomedical Engineering University of Toronto Toronto Ontario Canada; ^2^ Toronto General Hospital Research Institute University Health Network Toronto Ontario Canada; ^3^ Department of Chemical Engineering and Applied Chemistry University of Toronto Toronto Ontario Canada; ^4^ Terence Donnelly Centre for Cellular & Biomolecular Research University of Toronto Toronto Ontario Canada

**Keywords:** discovery and development, drug, organoids and organ‐mimetic systems, tissue engineering

## Abstract

Coronavirus disease 2019 (COVID‐19) has been a major global health concern since its emergence in 2019, with over 680 million confirmed cases as of April 2023. While COVID‐19 has been strongly associated with the development of cardiovascular complications, the specific mechanisms by which viral infection induces myocardial dysfunction remain largely controversial as studies have shown that the severe acute respiratory syndrome coronavirus‐2 can lead to heart failure both directly, by causing damage to the heart cells, and indirectly, by triggering an inflammatory response throughout the body. In this review, we summarize the current understanding of potential mechanisms that drive heart failure based on in vitro studies. We also discuss the significance of three‐dimensional heart‐on‐a‐chip technology in the context of the current and future pandemics.

**Translational Impact Statement**

The source of cardiac complications associated with COVID‐19 remains a subject of academic debate. In this comprehensive review, we describe potential mechanisms through which SARS‐CoV‐2 induces cardiac dysfunction, placing particular emphasis on recent advancements in heart‐on‐a‐chip systems for elucidating SARS‐CoV‐2 pathogenesis. We also highlight prospective developments in creating patient‐specific cardiac tissues, paving the way toward more advanced models for disease modeling and pharmacological discovery.

## 1 INTRODUCTION

Coronavirus disease 2019 (COVID‐19) is caused by a highly contagious virus, first reported in Wuhan in 2019 when multiple cases of pneumonia of unknown etiology were identified.^1^, ^2^ As of April 2023, more than 680 million cases of COVID‐19 have been confirmed across the globe. The virus sequence,^3^, ^4^, ^5^ protein structure,^6^, ^7^ and transcricptome^8^ became available in early 2020, which soon led to its identification as the severe acute respiratory syndrome coronavirus‐2 (SARS‐CoV‐2). The virus infects host cells by binding to angiotensin‐converting enzyme‐2 (ACE2),^9^, ^10^, ^11^ and subsequent activation of anti‐viral responses causes cellular damage. The lungs are its primary target, as it spreads through respiratory droplets, causing a wide spectrum of syndromes, including mild upper respiratory tract illness, severe viral pneumonia, and pulmonary fibrosis.^12^, ^13^ As the COVID‐19 pandemic progressed, reports of unexpected extrapulmonary damage emerged, with more detrimental injury to organs with high ACE2 receptor expression, including the heart and endothelial cells.^14^, ^15^ This observation is consistent with the high prevalence of cardiovascular complications observed in clinical settings, including cardiomyopathy, myocardial infarction, arrhythmias, hypercoagulation, and myocarditis.^16^, ^17^ While these data suggest a close association between SARS‐CoV‐2 infection and cardiovascular complications, the underlying mechanisms remain elusive.

Given the challenges in obtaining heart biopsies from COVID‐19 patients during or after the infection due to ethical and logistical considerations, alternative models including two‐dimensional (2D) cell cultures and animal models remain the gold standard for researching disease pathogenesis and screening drug responses. While cell lines may replicate viral entry and subsequent cytopathic effects, there is a growing understanding that the current methodologies may not adequately recapitulate drug responses and disease pathogenesis in humans. Simple cell line cultures often lack cell–cell and cell‐extracellular matrix (ECM) interactions, shear stress, and immune components, which results in epigenetic/functional deviation from native tissues and their natural interactions with the virus. Animal models also pose limitations, such as interspecies variations and resistance to SARS‐CoV‐2 infection, which can impact drug metabolism,^18^ viral replication kinetics,^19^ and disease pathogenesis,^20^ rendering it challenging to generalize findings to humans. For example, the antiviral properties of hydroxychloroquine were observed in non‐human primate model, but its efficacy did not translate to human clinical trials. This discrepancy can be attributed to the absence of cathepsin L, a crucial enzyme required for viral internalization into human lung cells.^21^ Therefore, to better understand organ dysfunction in the context of viral infection, there is an urgent need for a more physiologically relevant and reliable platform that adequately reflects clinical settings.

In the past 15 years, organ‐on‐a‐chip technology has emerged as a new addition to the in vitro systems that support the investigation of various aspects of human disease and pathophysiology. The core technology that advanced the organ‐on‐a‐chip field was microfabrication, a manufacturing process that enables the transfer of micro‐structures or micro‐patterns to devices with the aid of photolithography, etching, and deposition. As microfabrication techniques provide a dynamic spatial arrangement of materials at millimeter to micrometer precision, the technology was soon coupled with tissue engineering to reproduce key tissue microenvironment features, which gave rise to the organs‐on‐a‐chip and microphysiological systems. Concurrently, the convergence of induced pluripotent stem cell (iPSC) technology^22^ enabled the generation of functional cardiomyocytes that display phenotypic features closely resembling those of the human heart.^23^, ^24^ This facilitated a shift away from the use of immortalized cell cultures and many other animal‐derived cardiomyocytes for biological investigations. The increased complexities in heart‐on‐a‐chip systems provide unprecedented levels of organ‐level functions and recapitulate tissue‐specific responses, which marks a significant improvement over conventional 2D systems.

During the COVID‐19 outbreak, organ‐on‐a‐chip systems emerged as a valuable tool for elucidation of the pathophysiology of and host responses to SARS‐CoV‐2 infection. These systems provided insights into the mechanisms through which SARS‐CoV‐2 induces dysfunction in various organs, including the lung,^25^, ^26^, ^27^, ^28^ intestine,^29^, ^30^ and brain.^31^ Organ‐on‐a‐chip technology has expanded beyond academic research settings, fueled by the imperative to understand SARS‐CoV‐2 pathogenesis and to identify drug candidates to treat COVID‐19 patients. For instance, these models assisted in the identification of potential therapeutic targets that mediate tissue damage, which were later tested in clinical trials.^26^ Moreover, lung‐on‐a‐chip developed by both Emulate Inc. and MatTek Life Science were selected by Food and Drug Administration to screen potential therapeutics against COVID‐19 infection.^32^ The increasing demand for human‐mimetic systems has prompted the exploration of organ‐on‐a‐chip systems as a viable alternative to animal testing, and effectively bridging the preclinical testing phase to clinical trials. This review article aims to provide an overview of cardiac dysfunction in COVID‐19 patients and to summarize the current understanding of potential mechanisms that drive heart failure. It also underscores the growing significance of heart‐on‐a‐chip technology in the context of the ongoing pandemic and its potential implications in preparation for future pandemics.

## 2 IMPACT OF SARS‐COV‐2 ON HEART FUNCTION

Unlike the four common cold coronaviruses (HCoV‐229E, HCoV‐NL63, HCoV‐OC43, and HCoV‐HKU1) that do not cause cardiac complications, SARS‐CoV‐2 demonstrates a tropism to the heart. In early reports from Wuhan, an unexpected number of patients hospitalized with respiratory infections had elevated levels of high‐sensitivity troponin I, a marker that is closely associated with myocardial injury.^2^ In fact, cardiac dysfunction with increased levels of serum troponins is the most described COVID‐19‐associated abnormality, reported in 8%–20% of patients in multiple cohort studies.^1^, ^17^, ^33^ Several other blood markers, including the highly sensitive C‐reactive protein, N‐terminal pro‐B‐type natriuretic peptide (NT‐proBNP), creatine kinase‐myocardial band (CKMB), soluble IL‐1 receptor‐like 1,^34^ and miRNAs,^35^, ^36^ have also been studied in the context of cardiovascular complications in COVID‐19 patients.^37^ During the course of the COVID‐19 pandemic, it soon became apparent that elevated levels of cardiac injury markers are associated with a diverse spectrum of cardiovascular manifestations, including arrhythmia, myocardial infarction, and myocarditis.^17^ The adverse impact of SARS‐CoV‐2 on cardiac function underscores the urgency of understanding the underlying mechanisms of COVID‐19‐induced cardiac injury and paving the way toward development of novel therapeutic interventions to mitigate the risk of heart failure. Below, cardiac complications are grossly classified as “virus‐induced heart failure” and “virus‐associated heart failure” to distinguish between mechanisms that potentially drive SARS‐CoV‐2 pathogenesis. In most cases however, cardiac complications are caused as a result of severe inflammation, that is, they are virus‐associated.

### 2.1 Virus‐induced heart failure

COVID‐19‐induced heart failure is characterized by a change in functional cardiac output that is independent of systemic inflammation. In addition to being a respiratory disease, SARS‐CoV‐2 infection is also associated with endothelial cell dysfunction, with loss of junctional complexes between cells, leading to increased permeability of the capillary endothelium.^35^, ^38^ This enables virus access to several vital organs, including the heart. In the heart, the virus targets ACE2 receptors,^5^, ^10^, ^39^, ^40^, ^41^, ^42^ and virus enters the cells with the aid of transmembrane protease serine 2 (TMPRSS2), neuropilin‐1, and/or cathepsin L (CTSL).^9^, ^43^, ^44^, ^45^, ^46^, ^47^ It is important to emphasize that TMPRSS2 in the heart exhibits significantly lower expression than that in lungs,^40^ suggesting that CTSL may compensate for the absence of TMPRSS2 to mediate viral entry.^48^ Indeed, a study found that cardiomyocytes are less susceptible to SARS‐CoV‐2 infection compared to TMPRSS2‐positive Caco‐2 cells.^46^ This finding suggests that the TMPRSS‐2‐dependent endolysosomal route of entry is more favorable than CTSL‐dependent internalization, and that high local viral load and prolonged viral exposure may be necessary to successfully infect cardiomyocytes. The role of furin protease in SARS‐CoV‐2 internalization has also been explored, however, pharmacological intervention to limit viral replication using furin inhibitors has shown conflicting effects.^42^, ^45^ Considering the various mechanisms by which SARS‐CoV‐2 can infect cardiomyocytes, it comes as no surprise that multi‐organ autopsy studies identified the presence of SARS‐CoV‐2 viral RNAs in the cardiac tissue, suggesting SARS‐CoV‐2 tropism for the heart or other side populations such as pericytes.^49^, ^50^, ^51^

**FIGURE 1:**
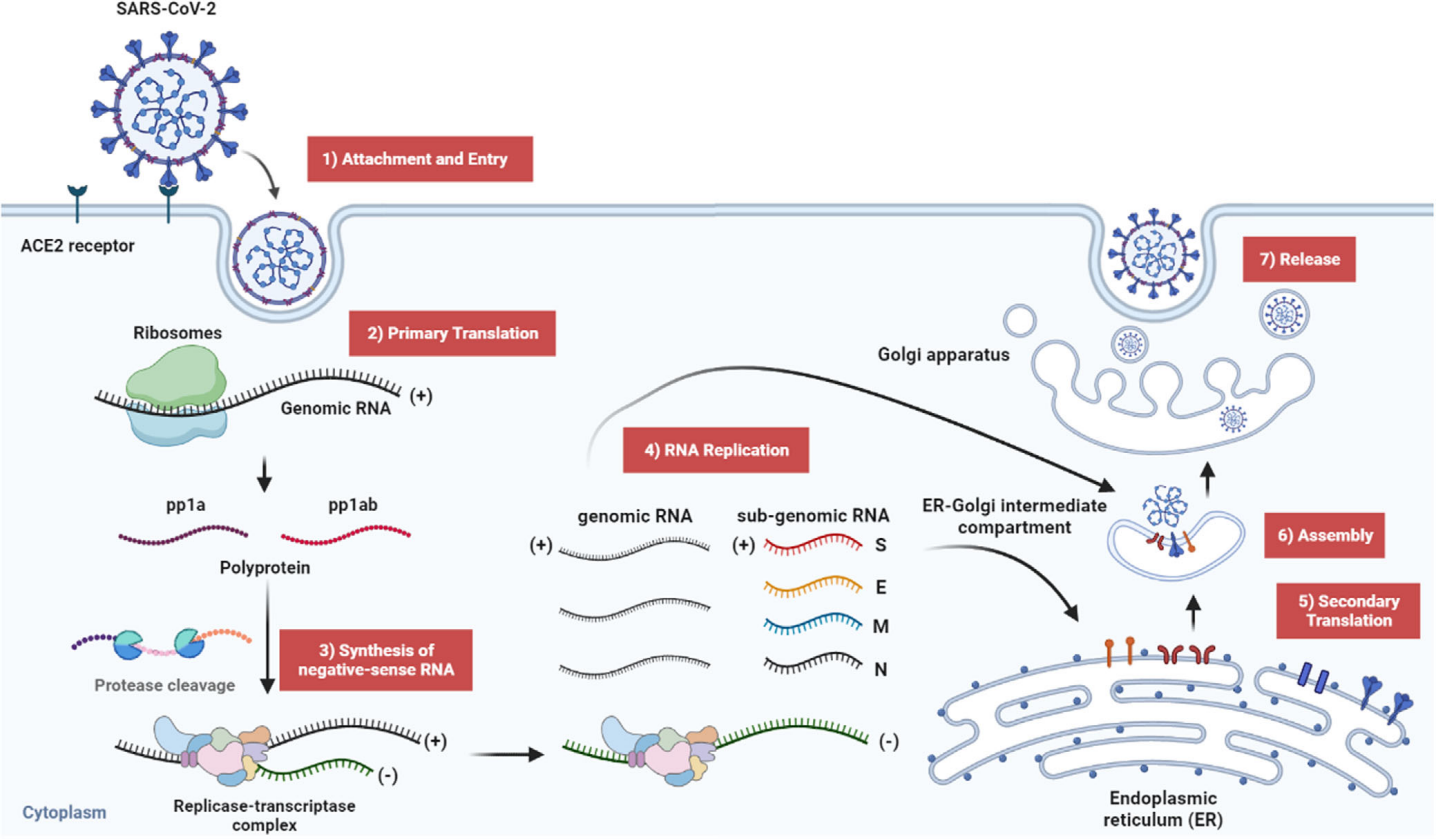
The coronavirus life cycle. The initial point of contact between SARS‐CoV‐2 and the host cell is the binding of the viral spike proteins to the ACE2 receptor, facilitating viral entry. Following the internalization, the viruses release their positive‐sense genomic RNA into the cytoplasm, which utilize the host's ribosomes to synthesize two polyproteins (pp1a and pp1ab). These polyproteins are subjected to proteolytic cleavage, giving rise to the replicase‐transcriptase complex composed of 16 nonstructural proteins. Among them, the RdRp orchestrates the generation of a complementary negative‐sense RNA strand from the viral genome. Using these negative‐sense RNA templates, the RdRp synthesize new positive‐sense genomic RNA copies, along with sub‐genomic RNAs that encode for structural proteins (S: spike, E: envelope, M: membrane, and N: nucleocapsid). Sub‐genomic RNAs are then translated into structural proteins at ribosomes located at the endoplasmic reticulum (ER). Following translation, the viral proteins are assembled within the ER‐Golgi intermediate compartment. These newly formed viral particles are transferred to the Golgi apparatus for packaging and are released from the host cell. This figure was created with the assistance of www.Biorender.com. ACE2, angiotensin‐converting enzyme‐2; SARS‐CoV‐2, severe acute respiratory syndrome coronavirus‐2.

Upon internalization, the virus discharges its positive‐sense single stranded RNA into the cytoplasm (Figure 1). The ribosomes then read the two large open reading frames (ORF1a and ORF1b) within the viral RNA, synthesizing two polyprotein precursors (pp1a and pp1ab). Proteolytic cleavage of these polyproteins yields the replicase‐transcriptase complex, compromising 16 nonstructural proteins, including the pivotal RNA‐dependent RNA polymerase (RdRp). RdRp is responsible for the synthesis of a negative‐sense RNA strand complementary to the viral genome, serving as a blueprint for both genomic and sub‐genomic RNA strands that encode structural and accessory proteins. Following the transcription of these negative‐sense RNA templates, the RdRp generates new positive‐sense RNA copies of the viral genome. Concurrently, newly formed positive‐sense sub‐genomic RNAs are translated into structural proteins, including the spike, membrane, nucleocapsid, and envelope proteins, by ribosomes attached to the endoplasmic reticulum (ER). After the translation, these proteins are transported to the ER‐Golgi intermediate for the final assembly with the genomic RNA. Finally, the newly assembled viral particles are transported to Golgi apparatus, where viral particles are packaged and released from the host cells by exocytosis, perpetuating the cycle of infection.

**FIGURE 2:**
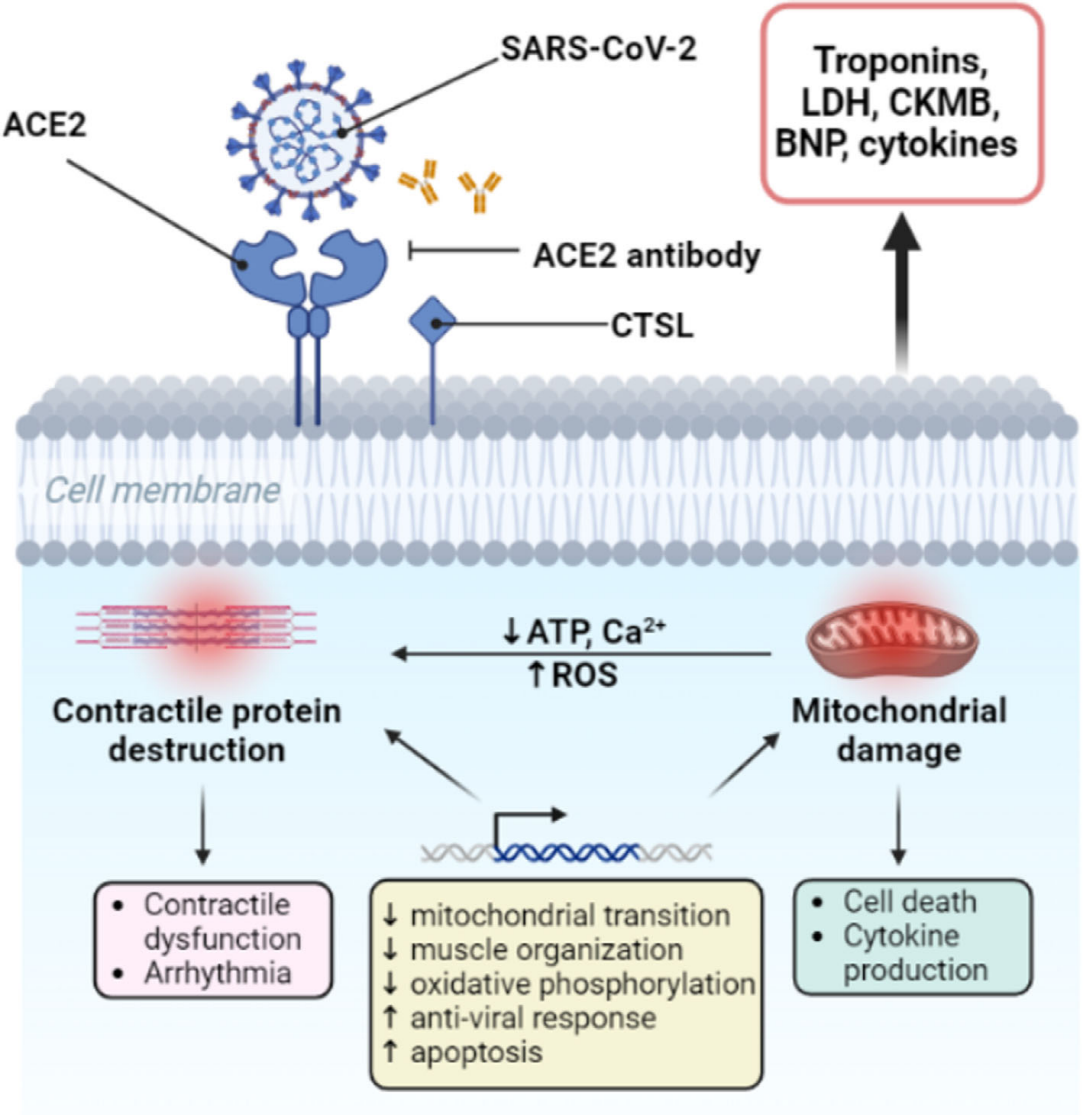
Key direct mechanisms of virus‐induced cardiac dysfunction. Infection by SARS‐CoV‐2 is initiated by the binding of spike proteins to ACE2 receptors and facilitated entry into the host cells by CTSL. Following internalization, the virus releases its genetic material, leading to significant transcriptional changes. These changes lead to mitochondrial damage and destruction of contractile proteins, culminating in heart dysfunction and cellular death. This figure was created with the assistance of www.Biorender.com. ACE2, angiotensin‐converting enzyme‐2; CTSL, cathepsin L; SARS‐CoV‐2, severe acute respiratory syndrome coronavirus‐2.

Human iPSC‐derived cardiomyocytes have been utilized to study the mechanisms identifying cardiomyocyte‐specific infection by SARS‐CoV‐2^52^, ^53^ usually when the virus is applied at relatively high multiplicity of infection (MOI). Under these conditions, SARS‐CoV‐2 exhibits a strong affinity toward iPSC‐derived cardiomyocytes, and leads to heart muscle fragmentation with loss of force production and cession of spontaneous contraction (Figure 2).^46^, ^53^ Consistent with other cell lines, the main cause of cardiac dysfunction appears to be the binding of SARS‐CoV‐2 to ACE2 and other co‐receptors.^54^ Evidence suggests that ACE2 knockout in WTC11C human cardiomyocytes or pretreatment of cardiomyocytes with an ACE2/CTSL inhibitor^46^ significantly decreases viral presence and apoptosis in the infected cells, flagging ACE2 and CTSL as potential therapeutic targets to prevent COVID‐19 cardiac complications (Figure 2). Infected cardiomyocytes also exhibited decreased expression of sarcomeric (*MYL2* and *MYL6*) and troponin (*TNNT2* and *TNNC1*) genes, which correlated with the dissolution of contractile machinery.^45^ These findings align with the significant myofibril abnormalities and clear evidence of damage to the sarcomeric banding in COVID‐19 autopsy samples.^45^, ^55^, ^56^

It is important to emphasize that a significant fraction of the heart is populated by non‐cardiomyocyte cells. Studies have revealed that cardiomyocytes only constitute 31% of the overall heart cell population, and it is being increasingly appreciated that non‐cardiomyocytes, including endothelial cells (43%), fibroblasts (15%), and resident macrophages (5%), are important regulators of heart homeostasis.^57^ Therefore, models that only use cardiomyocytes to understand COVID‐19‐associated heart pathology not only overestimate the interaction with cardiomyocytes and underestimate the impact of the virus on other cell types, but it also fails to consider critical crosstalk within the heart. Resident macrophages, for instance, have been shown to prevent fibrosis,^58^ facilitate electrical conduction in the atrioventricular node, contribute to tissue remodeling,^59^ and eliminate defective mitochondria.^60^ Therefore, studies must aim to characterize interactions of SARS‐CoV‐2 with the non‐cardiomyocyte population. Specifically, coculture systems are critical to deepen the understanding of COVID‐19 pathology. Several studies have indicated that human endothelial cells and fibroblasts exhibit negligible or no levels of ACE2 receptors, rendering them unfavorable for productive viral replication as compared to cardiomyocytes.^45^, ^61^, ^62^ This finding was corroborated by a recent study that employed human iPSC‐cardiomyocytes cocultures with fibroblasts and macrophages; viral genomes were not detected in either fibroblasts or macrophages.^63^ Moreover, single‐cell atlas of the human heart has unveiled that pericytes display elevated levels of ACE2, that may serve as the target for SARS‐CoV‐2.^64^, ^65^ The in‐depth cell–cell interaction analysis also demonstrated an extensive connection between endothelial cells and pericytes, indicating a critical role of pericytes in preserving endothelial cell functionality. Remarkably, the administration of human recombinant soluble ACE2 to blood vessel organoids composed of endothelial cells and pericytes reduced viral infection.^66^ This finding suggests that the infection of pericytes could potentially contribute to the observed cell death, and targeting ACE2 on pericytes may represent a promising strategy to effectively mitigate the associated damage. Nevertheless, extended exposure of endothelial cells and fibroblasts to SARS‐CoV‐2 has been demonstrated to trigger cellular death and viral replication, thereby highlighting virus capacity to induce cellular toxicity via an ACE2‐independent mechanism.^45^ This may occur through various mechanisms, such as attachment of extracellular vimentin to SARS‐CoV‐2,^67^ cleavage of NF‐κB essential modulator by a main protease of SARS‐CoV‐2,^68^ SARS‐CoV‐2 nonstructural protein‐induced vascular dysfunction,^69^ activation of toll‐like receptor 4 (TLR‐4)‐mediated nuclear factor kappa B (NF‐κB) signaling,^70^ or α_v_β3 integrin‐mediated endocytosis.^71^

The presence of SARS‐CoV‐2 not only impairs the mechanical functions of the heart, but also facilitates several electrophysiological abnormalities. In a prospective international study involving sites in 69 countries, more than 50% of COVID‐19 patients had an abnormal echocardiogram with left/right ventricular abnormalities, myocardial infarction, myocarditis, and Takotsubo cardiomyopathy.^72^ Hospitalized COVID‐19 patients also exhibited varying degrees of QT interval prolongation, with some progressing to *torsade de pointes*, a type of ventricular tachycardia characterized by a twisting of the QRS complex.^73^ However, it remains unclear whether these electrophysiological irregularities arose from direct interaction with SARS‐CoV‐2 or from systemic inflammation. Using a microelectrode array, Marchiano et al. observed propagation of the field potential duration in SARS‐CoV‐2‐infected human iPSC‐derived cardiomyocytes, which represents an in vivo alternate of the QT interval measured by electrocardiography.^74^ Additionally, SARS‐CoV‐2 infection has been shown to reduce the spontaneous beating rate, lower depolarization spike amplitude, and decrease electrical conduction velocity in cardiomyocytes, suggesting that direct virus–cardiomyocyte interactions and subsequent electrophysiological dysfunction directly creates a substrate for arrhythmias as observed in COVID‐19 patients.^75^

In addition to the observed electrophysiological alterations in the heart, SARS‐CoV‐2 infection leads to various transcriptional changes. RNA‐sequencing studies have shown downregulation of genes related to cardiac muscle organization,^45^, ^61^, ^76^ mitochondrial translation,^53^, ^63^ and oxidative phosphorylation in infected cardiomyocytes (Figure 2). As mitochondria play a critical role in providing energy and maintaining calcium homeostasis in the heart, decreased expression of genes related to mitochondrial translation and oxidative phosphorylation may lead to decreased energy production, reactive oxygen species (ROS) imbalance, and arrhythmia. In contrast, SARS‐CoV‐2 infection upregulated transcriptional changes related to apoptosis^46^, ^77^ and anti‐virus immune response.^45^, ^53^, ^63^, ^76^ Among the reported anti‐virus immune responses, interferon‐related genes were significantly enriched in most studies, which may further promote cardiomyocyte dysfunction. As such, SARS‐CoV‐2 infection leads to an array of alterations in cardiomyocyte function, which act synergistically to exacerbate heart failure.

### 2.2 Virus‐associated heart failure

**FIGURE 3:**
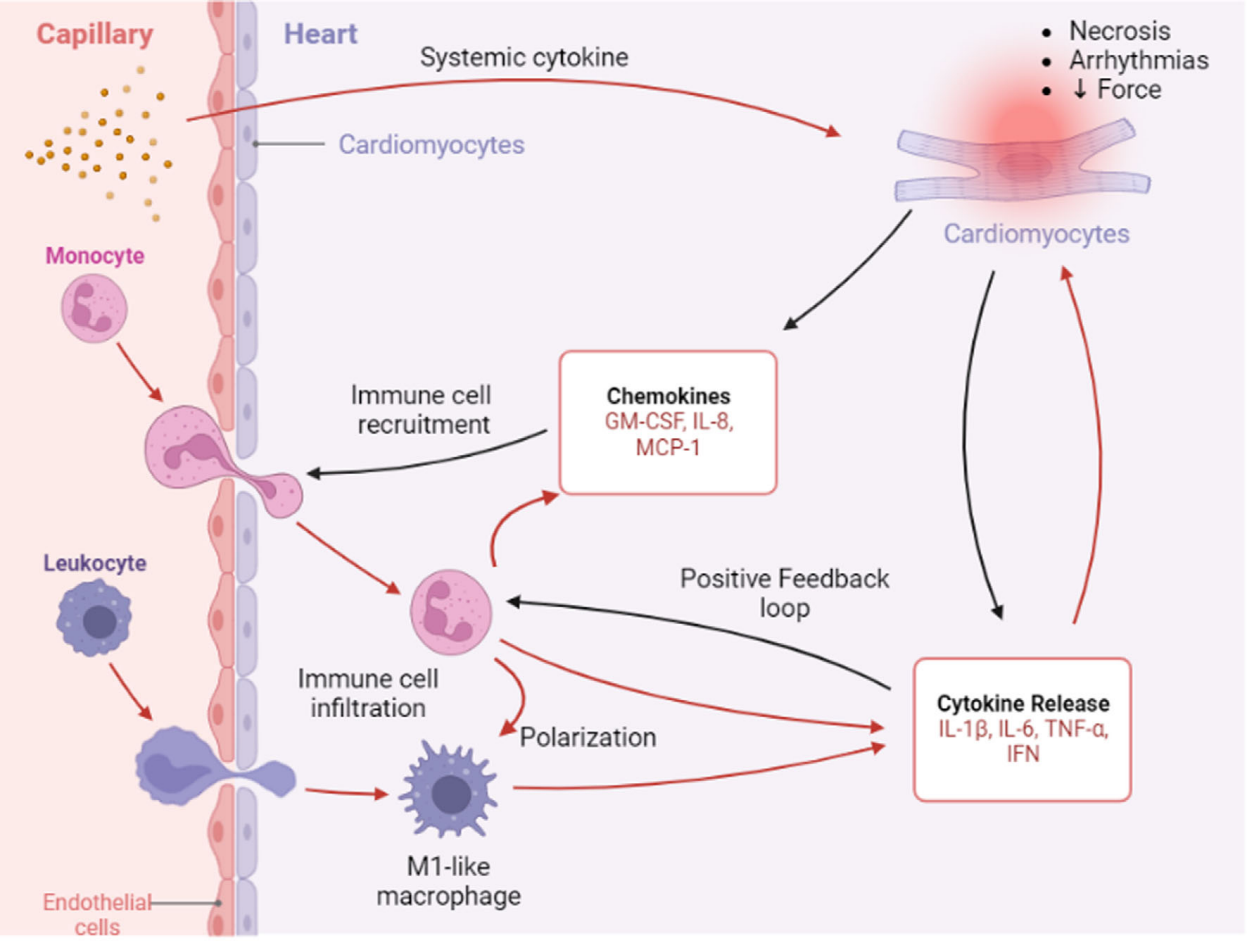
Key mechanisms of inflammation‐driven virus‐associated cardiac dysfunction. Systemic inflammation induces cardiomyocyte damage, triggering the release of chemokines that recruit immune cells to the site of injury. Infiltrated monocytes can be polarized into M1‐like macrophages that release proinflammatory cytokines. These cytokines not only establish a positive feedback loop to amplify the release of cytokines and chemokines, but also contribute to a synergistic amplification of tissue inflammation. This figure was created with the assistance of www.Biorender.com.

Whereas damage of cardiomyocytes via direct infection occurs only rarely in the clinic, virus‐associated systemic inflammation is the prevalent mechanism of cardiac complications. COVID‐19, like other viral outbreaks, has been linked to an increased incidence of cardiovascular events, which directly correlate with the severity of inflammation. Myocarditis is an emerging concern of COVID‐19, as it can cause permanent and irreversible damage to the heart muscle, even after the initial infection has resolved. Uncontrolled SARS‐CoV‐2 infection can trigger cytokine storm, which is mediated by pro‐inflammatory cytokines and chemokines, including IL‐2, IL‐4, IL‐6, IFN‐γ, and TNF‐α, produced during innate anti‐viral immune responses^2^, ^78^, ^79^ (Figure 3). Elevated systemic levels of cytokines trigger endothelial cells to adopt an inflammatory phenotype, characterized by the expression of cytokines/chemokines as well as vascular adhesion molecules.^35^ Such endothelial cell activation promotes leukocyte recruitment and transmits intracellular signals, leading to further activation and infiltration of innate immune cells into the cardiac parenchyma, causing myocardial inflammation, also known as myocarditis. SARS‐CoV‐2‐infected cardiomyocytes can also secrete CCL2 (also known as MCP‐1), which attracts immune cells to further exacerbate tissue injury by releasing pro‐inflammatory cytokines.^61^, ^76^ The post‐mortem analysis of COVID‐19 patients has revealed the presence of inflammatory infiltration of CD163+, CD11b+, and CD68+ macrophages, as well as CD3+ and CD4+ T‐lymphocytes in the heart tissues.^50^, ^51^, ^80^, ^81^, ^82^ Indeed, the highest macrophage accumulation in COVID‐19 patients was found in sites of cardiomyocyte injury, indicating that increased immune cell infiltration along with systemic inflammatory mediator levels raise the risk for severe illness and mortality.

While the incidence of acute myocarditis is rare,^33^, ^83^, ^84^, ^85^, ^86^ compromising approximately 0.2%–0.3% of reported COVID‐19 cases,^87^ it poses a serious risk for cardiovascular complications. More specifically, 39% of patients with COVID‐19‐associated myocarditis developed a fulminant form of heart failure and 70% of them were admitted to an intensive care unit (ICU). Among all COVID‐19‐associated deaths, 7% were attributed to myocarditis and 78.7% of COVID‐19 patients whose death was attributed to myocarditis exhibited some form of ventricular arrhythmia, highlighting distinct feature of SARS‐CoV‐2 myocarditis in causing extensive cardiac involvement.^88^, ^89^, ^90^ The pathogenic process of viral myocarditis can be divided into three main phases: an acute phase during which the virus enters the cells and activates the innate immune cells, which can last 1–7 days, a subacute phase which can last 1–4 weeks, and a chronic phase that can last from months to years. SARS‐CoV‐2‐mediated myocarditis is generally considered acute, as the cardiac injury is primarily driven by inflammation secondary to cytokine storm. The subsequent myocardial inflammation leads to infiltration of immune cells into the heart, which further promotes a hyperinflammatory microenvironment that can result in a wide spectrum of heart dysfunctions. Recent studies also highlighted the prolonged risk for cardiovascular diseases in patients with a history of COVID‐19 infection, particularly in individuals with long‐COVID, an illness that can persist beyond the acute phase of infection.^91^

A range of in vitro models featuring diverse cardiovascular cell types and immune cells have been used to shed light on how inflammatory responses indirectly exacerbate cardiac injury. Several studies utilizing human iPSC‐derived cardiomyocytes or human heart biopsies verified that infected cardiomyocytes could recruit immune cells by secreting or upregulating the gene expression of monocyte chemoattractant protein, CCL2.^61^, ^76^ Infiltrated immune cells, such as CCR2+ monocytes then polarize into the M1‐like phenotype, which is associated with proinflammatory properties that elevate genes associated with ROS and reduce expression of genes linked with cardiac function (Figure 3). In turn, this promotes apoptosis, cytolysis, and functional damage. An immuno‐cardiac coculture platform also demonstrated that recruited macrophages elevate cytokine levels (IL‐1β, IL‐6, and TNF‐α) and set off a vicious circle of inflammation, which contributes to a progressive feedback loop of immune activation and subsequent myocardial damage. Notably, treatment of cardiomyocytes with CCL‐2‐neutralizing antibodies or CCR2 (receptor of CCL2) inhibitors significantly attenuated monocyte recruitment to cardiomyocytes,^76^ suggesting that CCL2 serves as the principal mediator of monocyte migration upon SARS‐CoV‐2 infection.

In addition to the role of SARS‐CoV‐2 in the indirect activation of systemic immune responses, several studies have suggested that the virus can directly activate immune cells^92^, ^93^ and shift their canonical innate immune phenotype to a pro‐thrombotic phenotype.^94^ At the transcriptional level, monocytes isolated from COVID‐19 patients display upregulation of pathways associated with hemostasis, immune‐thrombosis, and platelet aggregation, which ultimately lead to thrombosis generation. Indeed, thrombotic incidents can manifest across diverse anatomical locations, including the pulmonary system, deep veins, and conceivably extending to cardiovascular system, leading to potentially life‐threatening myocardial infarction and heart failure.^95^, ^96^ When exposed to SARS‐CoV‐2, viruses bind to human macrophages/monocytes through TLR‐2 or TLR‐4, which trigger the assembly of the NLRP3 inflammasome, a complex of proteins that responds to both pathogen‐associated injury and cellular stressors.^93^, ^97^ SARS‐CoV‐2 can also infect monocytes via Fcγ receptors and monocytes undergo pyroptosis‐mediated activation of NLRP3 and AIM2 inflammasomes. Inflammasome activation leads to IL‐1β release, which can be greatly amplified through an autocrine loop or a positive‐feedback loop, resulting in an excessive and uncontrolled response, known as cytokine storm. Moreover, IL‐1β‐mediated activation of endothelial cells can lead to the downregulation of VE‐cadherin transcription, ultimately resulting in the loss of adherens junctions that are essential for the maintenance of barrier function.^98^ This, in turn, promotes the accumulation of immune cells in the heart and forms a hyperinflammatory microenvironment that carries a high burden of cytokine‐secreting monocytes and neutrophiles, which are strongly associated with severe disease outcomes. Thus, the synergistic interplay between feedback amplified immune cell recruitment and feedforward tissue damage can significantly intensify heart injury, particularly in response to the initial uncontrolled release of proinflammatory cytokines directed by IL‐1.

## 3 HEART‐ON‐A‐CHIP IN COVID‐19 RESEARCH

**FIGURE 4:**
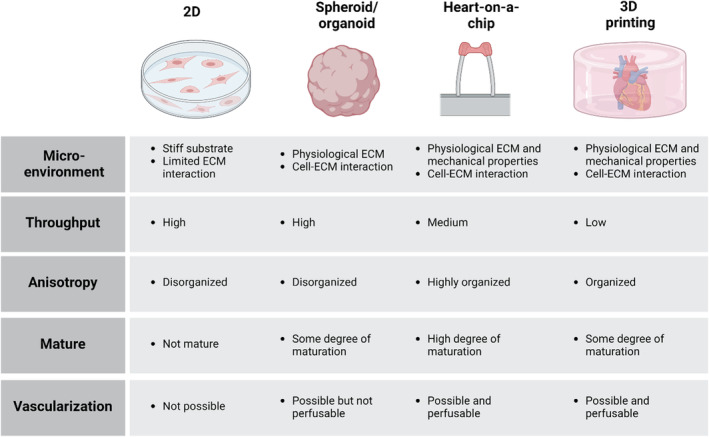
A summary of advantages and disadvantages of various cardiac models. The classification of existing methodologies and the resultant cellular phenotype can be primarily categorized into five key parameters: interaction of cells with their microenvironment, device throughput capacity, cellular anisotropy, cellular maturity, and the incorporation of vascular structures.

While 2D human iPSC‐derived cardiomyocytes have had a transformative impact on both fundamental and applied research, disorganized contractile arrangement and absence of a cardiac microenvironment may not result in proper function, which limits the interpretation and application of research findings (Figure 4). The natural geometry of the myocardium is three‐dimensional and highly anisotropic, necessitating methodologies that can spatially control cardiomyocyte geometry. In an effort to overcome the limitations inherent to traditional 2D systems, two distinct yet intertwined tissue engineering strategies have been explored for the generation of functional cardiac tissues. The first approach is the generation of cardiac spheroids or organoids, a three‐dimensional (3D) and self‐organized assemblies that spontaneously coalesce into spherical structures. It is important, however, to differentiate between the terms “spheroids” and “organoids” despite their frequent interchangeable usage. Cardiac spheroids are typically generated by integrating post‐differentiated cells constituting cardiomyocytes, cardiac fibroblasts, and endothelial cells akin to the cellular composition of the heart, while cardiac organoids emerge from the self‐aggregation of pluripotent stem cells and subsequently differentiating into cardiac cells.^99^, ^100^ These 3D constructs emulate a more native tissue architecture and facilitate intercellular communication between cardiomyocytes and other cell types, while simultaneously being amendable to high‐throughput operations, which presents a marked advantage over traditional monolayer cultures (Figure 4). However, spheroids and organoids generally lack the anisotropy of myocardium, necessitating methodologies that can spatially control cardiomyocyte geometry.

**FIGURE 5:**
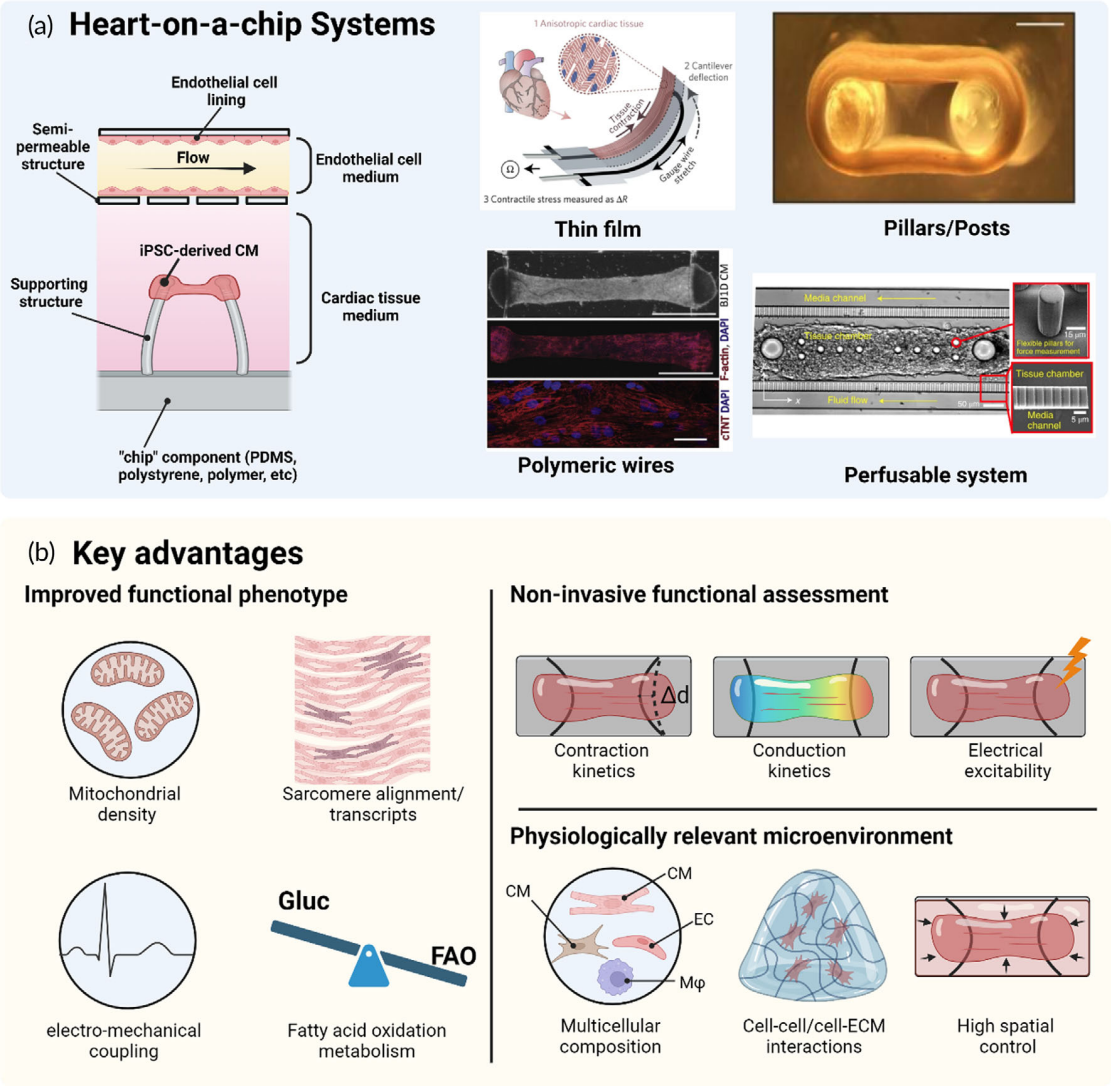
Heart‐on‐a‐chip system configurations and key advantages. (a) The heart‐on‐a‐chip systems are designed to incorporate “chip” components that provide spatial confinement for the human‐derived iPSC cardiac tissues. The system is complemented with supporting structures such as thin films,^117^ micro‐pillars/posts,^107^ and polymeric wires,^111^ which facilitates the self‐assembly of cardiac tissues into an oriented structure. Vascularized heart‐on‐a‐chip system with perfusion can simulate in vivo blood flow conditions.^124^ (b) Advantages of 3D tissue models over the traditional 2D systems. Cardiac tissues grown on the heart‐on‐a‐chip models exhibit enhanced functional phenotype, permit non‐invasive functional assessment, and provide physiologically relevant microenvironment that closely mimic the human myocardium. Reproduced with permission.^107^, ^111^, ^117^, ^124^ This figure was created with the assistance of www.Biorender.com. iPSC, induced pluripotent stem cell.

For this reason, microfabrication techniques have been harnessed to bioengineer tissue to the proper geometries and with appropriate microenvironments, that give rise to heart‐on‐a‐chip system. Similar to cardiac spheroids/organoids, heart‐on‐a‐chip models leverage the culture of human iPSC‐derived cardiomyocytes, often combined with other cell types, such as cardiac fibroblasts and endothelial cells, within the extracellular matrix, enclosed within micropatterned configurations (Figure 5a). The construction of heart‐on‐a‐chip platforms is commonly performed using soft lithography techniques, where micrometer‐scale structures are fabricated into biocompatible elastomeric material such as poly(dimethysiloxane) (PDMS), generally to serve as two anchor points for cylindrical tissue generation. However, due to the hydrophobic nature of PDMS, a broad spectrum of chemical substances and drugs have been observed to adsorb and absorb on such a material.^101^ Therefore, alternative materials such as polystyrene and synthetic polymers have been explored for standard “on‐chip” devices.^102^ Culturing human iPSC‐derived cardiomyocytes within micropatterned configurations alongside supporting structures, for example, micro‐posts/pillars,^103^, ^104^, ^105^, ^106^, ^107^ cantilevers,^108^, ^109^ elastomeric wires,^110^, ^111^, ^112^, ^113^, ^114^ or thin films^115^, ^116^, ^117^, ^118^, ^119^ has facilitated cardiac tissue modeling and promoted the self‐organization of tissues into oriented structures (Figure 5a). Cardiac tissues cultured in such a spatially confined environment display many  ultrastructural characteristics similar to those seen in mature myocardium. These features include the manifestation of physiological sarcomere length and alignment, the appearance of transverse tubules, the increased density of mitochondria, and a positive force–frequency relationship.^107^, ^111^, ^120^, ^121^ Numerous studies have demonstrated that the utilization of the 3D tissue cultured on heart‐on‐a‐chip systems leads to the development of cardiac tissues that closely resemble native human myocardium, as evidenced by improved electrophysiological properties,^122^ protein expression, and gene expression^107^, ^111^, ^120^ compared to cardiomyocyte cultured in monolayers (Figure 5b). Specifically, monolayer cultivation of iPSC‐derived cardiomyocytes fails to mature Ca^2+^ handling apparatus as evidenced by the low caffeine sensitivity and short action potential duration (APD) in comparison to 3D cultures.^112^ Integration of polymeric microchannel lined with perfusable endothelial cells or microfluidic conduits into established heart‐on‐a‐chip systems can also enable the formation of vascularized cardiac tissues.^108^, ^109^, ^123^, ^124^, ^125^ These systems can provide clinically relevant approaches for drug administration or the delivery of circulating immune cells, thereby improving the overall fidelity to in vivo conditions.

Such engineered 3D cardiac tissues and heart‐on‐a‐chip systems, present a promising avenue for the generation of functional cardiac tissues and have been instrumental in answering questions that cannot be readily answered with standard 2D cell models and cardiac spheroids/organoids (Figure 5b). For instance, supporting structures not only provide mechanical cues for the cardiac remodeling process, but also facilitate the measurement of cardiac tissue‐generated contraction forces by monitoring the displacement of the supporting structure (Figure 5b). Built‐in carbon electrodes can also be exploited to provide electrical pacing, which is needed to accurately analyze intracellular Ca^2+^ activities and conduction velocity.^111^ The non‐invasive assessment of cardiac tissue function and responses holds significant importance in the field of cardiac research, as it offers potential clinical translation for understanding the relationship between altered contraction patterns and Ca^2+^ kinetics with underlying cardiac diseases and abnormalities. COVID‐19 patients frequently present with ion channel abnormalities, reduced ventricular ejection fraction (LVEF), and a range of clinical manifestations. By incorporating heart‐on‐a‐chip systems, comprehensive investigations into the underlying pathophysiological mechanisms driving cardiac complications can be conducted, shedding light on the intricate interplay between disease progression and cardiac dysfunction.

More recently, 3D printing technology has emerged as a next‐generation approach to constructing heart models, with these methods leveraging the use of bioinks to embed cells. This provides enhanced control over structural design and facilitates the formation of vascularized tissues that are capable of implantation in vivo settings. 3D printing also allows for the generation of heart constructs mimicking the human size^126^ and multi‐scale complexity of vascular networks.^127^, ^128^, ^129^, ^130^ Alternatively, heart‐mimicking construct can also be prepared by de‐cellularizing heart with detergent, and re‐cellularizing with cardiac cells.^131^ However, it is important to note certain limitations associated with 3D printing that warrant careful consideration. First of all, an inherent trade‐off exists between the operational throughput and the biological complexity of the cardiac tissue models. Implementing 3D printing technique can result in significant cost, complexity, and low throughput, potentially hindering their broad commercialization, reproducibility, and clinical translation. Additionally, the bioinks used in these systems may not be suitable for long‐term culture, as the vasculature within the hydrogel has a tendency to collapse during extensive tissue remodeling. While this review will reference 2D models, spheroids/organoids, and 3D printing systems where relevant for comparative purposes, the primary focus will lie on the exploration of heart‐on‐a‐chip models. Below, we demonstrate the utility of heart‐on‐a‐chip models as an indispensable in vitro tool for modeling COVID‐19‐induced cardiovascular damage.

### 3.1 Recapitulating heart‐SARS‐CoV‐2 interactions using heart‐on‐a‐chip systems

**FIGURE 6:**
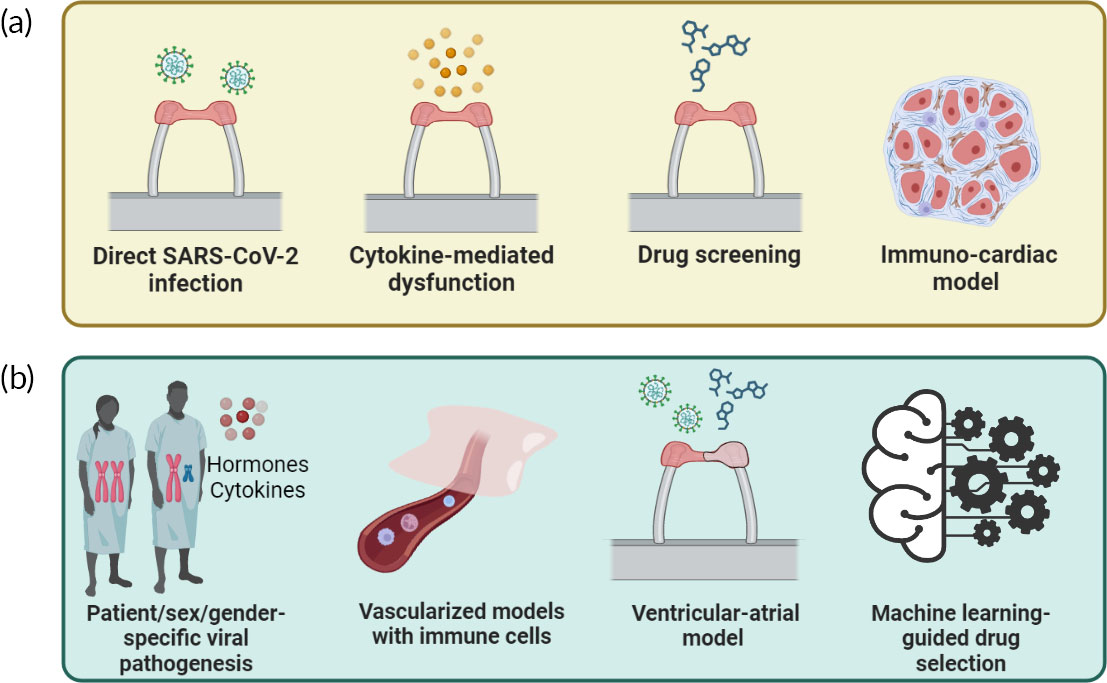
Current progress and opportunities in cardiac tissue engineering using heart‐on‐a‐chip systems. (a) During COVID‐19 pandemic, engineered heart tissues were exposed to SARS‐CoV‐2 or a combination of cytokines to induce disease phenotype, allowing for the investigation of underlying pathological mechanisms and drug screening. By modifying cell composition, heart‐on‐a‐chip systems can successfully model various cardiac conditions, including the generation of immune‐cardiac model for studying myocarditis within the context of viral infection. (b) A roadmap for future directions. Use of iPSC‐derived cardiomyocytes with distinct genetic mutations and sex‐specific cells to elucidate the impact of preexisting heart conditions, sex differences, and specific hormonal environment on heart failure. Advanced vascularized heart‐on‐a‐chip platforms will enable the integration of immune components and physiological dosing of viruses/drugs. Chamber‐specific cardiac tissues are vital, as ventricles and atrium exhibit distinctive drug responses. Finally, the improved scalability of these systems facilitates the use of machine learning to accelerate the drug discovery process. This figure was created with the assistance of www.Biorender.com. COVID‐19, Coronavirus disease 2019; SARS‐CoV‐2, severe acute respiratory syndrome coronavirus‐2.

Heart‐on‐chip models integrating iPSC‐derived cardiomyocyte and fibroblast cocultures have been utilized to generate contracting tissues and to simulate viral infection of cardiac tissue (Figure 6a). A number of reports using heart‐on‐a‐chip models have shown that viral infection of cardiac tissues causes functional alterations. For instance, Marchiano et al. used fibrin‐embedded engineered 3D cardiac tissues composed of human WTC11c iPSC‐derived cardiomyocytes and HS27a stromal cells.^74^ The cardiac tissues were suspended between silicon posts, where one post is rigid and another is flexible containing an embedded magnet, which is used to monitor contractile behavior through magnetic sensing. When the cardiac tissues are infected at a supraphysiological concentration at MOI of 10, they exhibited reduced contraction amplitude and frequency relative to the mock‐infected tissues. Additionally, infected tissues displayed decreased expressions of sarcomeric genes (MYL2, MYH6) and troponin T, which may underlie the loss of sarcomere organization and contractile properties. These findings align with a clinical decrease in LVEF measured in some COVID‐19 patients, particularly in those with severe or critical illness. The heart‐on‐a‐chip platforms also offer a distinct advantage in that they enable replication of the cardiac microenvironment. By incorporating macrophages into engineered cardiac tissue models, it becomes possible to explore whether the infection of these constructs with SARS‐CoV‐2 can replicate key features of COVID‐19 myocarditis, thereby offering valuable insights into the contribution of immune cells to the disease pathogenesis (Figure 6a).^63^ Specifically, Bailey et al. used heart‐on‐a‐chip featuring iPSC‐derived cardiomyocytes, fibroblasts, and macrophages, integrated within a collagen‐Matrigel hydrogel sandwiched between two flexible PDMS posts. Upon infection with SARS‐CoV‐2 infection at MOI of 0.1, CD68 macrophages exhibit marked preferential accumulation in the vicinity of the viral nucleocapsid. This accumulation could instigate a hyperinflammatory microenvironment, which, in turn could amplify tissue damage, as evidenced by the attenuated contraction amplitude and the decreased contraction and relaxation speed. It is important to highlight that viral nucleocapsid protein preferentially accumulates at the periphery of cardiac tissues, as the extracellular matrix may act as a physical barrier hindering viral attachment and impeding viral diffusion into host cells. This highlights the limitations of 2D systems in accurately assessing virus interactions with cardiomyocytes, raising questions about the translation relevance of such models in the understanding of heart dysfunction.

In addition to investigating the direct myocardial interactions with SARS‐CoV‐2, it is critical to investigate the impact of elevated cytokines using heart‐on‐a‐chip systems. The inflammatory response is of particular concern, as cytokines can pass through the extracellular matrix and diffuse throughout the heart to mediate injury. Mills et al. generated cardiac organoids composed of iPSC‐derived cardiomyocytes, fibroblasts, and endothelial cells embedded in collagen I‐Matrigel hydrogel and confined between two PDMS poles in a 96‐well format. This high‐throughput and high‐content screening platform enables the assessment of 40 different combinations of cytokines (LPS, IL‐1β, IL‐6, IL‐17A, G‐CSF, poly(I:C), TNF‐α), with the aim of identifying the “cytokine cocktail” responsible for cardiac dysfunction (Figure 6a). A combination of IFN‐γ, IL‐1β, and poly(I:C) prolonged relaxation time decreased the beating rate, key pathological signatures of diastolic dysfunction. This implies that cardiac injury can be elicited independently of direct cardiomyocyte interaction and that the cytokines triggered by SARS‐CoV‐2 infection may constitute a pivotal mediator of the cardiac damage experienced by COVID‐19 patients. Using single‐cell nuclei RNA sequencing and phosphoproteomics, signal transducer and activator of transcription 1 (STAT‐1) and bromodomain‐containing 4 (BRD‐4) were identified as potential therapeutic targets for treating cytokine‐induced heart complications. When the corresponding inhibitors were screened, JAK/STAT inhibitors (baricitinib and ruxolitinib) or CDK8 inhibitors displayed limited recovery of cardiac functions, while a BET inhibitor (ICNB054329) prevented inflammation‐induced diastolic dysfunction without affecting tissue contraction. While this study primarily investigated a subset of cytokines associated with diastolic dysfunction, the use of state‐of‐the‐art proteomics and sequencing methods to explore cytokine mixtures that cause other symptoms of COVID‐19‐associated heart failure, such as arrhythmia and systolic dysfunction, may facilitate identification of potential therapeutic targets.

### 3.2 Heart‐on‐a‐chip systems to evaluate repurposed and preclinical drugs

During the COVID‐19 pandemic, many pre‐existing FDA‐approved drugs were repurposed as anti‐viral therapeutics or prophylactics (Table 1). However, unexpected cardiotoxicity elicited by these drugs increased the risk for cardiovascular complications, and many drugs were withdrawn as their therapeutic benefit did not outweigh their known risks. During the initial stages of the pandemic, hydroxychloroquine and remdesivir were considered promising treatment options. Yet, evidence of serious safety issues accumulated, including severe arrhythmias, QT‐interval prolongation, *torsades de pointes*, and sudden death in COVID‐19 patients treated with hydroxychloroquine, often in combination with azithromycin.^132^, ^133^ To assess the potential risk of drug‐induced cardiac toxicity in COVID‐19 patients, heart‐on‐a‐chip platforms could serve as a platform for systemic evaluation of repurposed or novel drug candidates before they are entered into clinical trials (Figure 6a). Ideally, such testing should be performed in the presence of inflammatory cytokines. For instance, Charrez et al. used a heart‐on‐a‐chip system, consisting of WTC human iPSC‐derived cardiac tissues separated by two adjacent PDMS perfusable channels subjected to physiological shear stress of 6 dyn/cm^2^, to simulate in vivo drug transport and investigate the effects of hydroxychloroquine and azithromycin on cardiac tissue function. This heart‐on‐a‐chip platform proved instrumental in predicting clinical arrhythmias characterized by QT prolongation and rhythmic instability in cardiac tissues subjected to hydroxychloroquine treatment.^134^ The combined treatment with hydroxychloroquine and azithromycin also imparted a synergistic effect on QT interval duration, thereby corroborating the rationale behind their removal from the list of drugs authorized for emergency use. In another study, Xu et al. constructed cardiac bundles consisting of human embryonic stem cell‐derived cardiomyocytes encapsulated in fibrinogen‐Matrigel mixture, that is anchored to flexible nylon frames. This setup was utilized to investigate the potential cardiotoxic effects of various anti‐viral compounds, such as amilimod, remdesivir, ritonavir, and loponavir. At clinically relevant concentrations, these drugs were shown to impair tissue contractile function and downregulate gene expresions related to sarcomere organization and ion homeostasis.^52^ This study is noteworthy in that it both identified drugs that alleviate the cardiotoxicity of the aforementioned drugs, but also discussed the need for additional drugs that can mitigate the cytotoxic effects of anti‐viral drugs while preserving their anti‐viral potency. Taken together, the use of heart‐on‐a‐chip systems prior to clinical trials enables rapid evaluation of the cardiac safety of re‐purposed drug candidates before administering them to the patients.

**TABLE 1:** List of candidate COVID‐19 drugs screened in cardiomyocytes.

Mechanism of action	Drug	Model	Pathological induction	In vitro finding
CDK inhibition	SEL‐120‐34A	3D^137^	Cytokine	Does not improve contraction force but improves contraction rate.
	BI‐1347	3D^137^	Cytokine	Does not improve contraction force but improves contraction rate.
	Flavopiridol	3D^137^	Cytokine	Decreases force and 50% activation time.
BET inhibition	INCB054329	3D^137^	Cytokine	Blocks cytokine‐induced cardiac diastolic dysfunction without affecting force.
	JQ‐1	3D^137^	Cytokine	Blocks cytokine‐induced cardiac diastolic dysfunction in specific cell lines.
	ABBV‐744	3D^137^	Cytokine	Exacerbates diastolic dysfunction.
JAK/STAT inhibition	Baricitinib	3D^137^	Cytokine	Prevents TNF‐induced systolic dysfunction.
	Ruxolitinib	3D^137^	Cytokine	Does not improve contraction force.
		2D^45^	Cytokine/SARS‐CoV‐2	Inhibits IFN response.
Blocks viral entry	Hydroxychloroquine	3D^134^	N.A.	Causes QT prolongation and arrhythmia.
Antibiotic	Azithromycin	3D^134^	N.A.	Causes arrhythmia.
ACE2 inhibition	ACE2 antibody	2D,^45^, ^46^ 3D^63^	SARS‐CoV‐2	Decreases proinflammatory expression and viral copies in cardiomyocytes.
	miR‐200c	2D^169^	N.A.	Decreases ACE2 protein levels.
CCL2 inhibition	CCL2 antibody	2D^76^	SARS‐CoV‐2	Decreases monocyte recruitment to cardiomyocytes.
CCR inhibition	RS504393	2D^76^	SARS‐CoV‐2	Decreases monocyte recruitment to cardiomyocytes.
TMPRSS2 inhibition	Aprotinin	2D^45^	SARS‐CoV‐2	Does not inhibit viral infection.
	Camostat	2D^45^	SARS‐CoV‐2	Does not inhibit viral infection.
RdRp inhibition	Remdesivir	2D^45^, ^46^	SARS‐CoV‐2	Inhibits viral replication but induces marked toxicity in cardiomyocytes.
		3D^63^	SARS‐CoV‐2	Inhibits viral replication and maintains troponin organization.
		2D, 3D^52^	N.A.	Induces apoptosis and sarcomeric disarray. Weakens contractile forces.
Cathepsin‐L inhibition	ALLM	2D^46^	SARS‐CoV‐2	Inhibits viral replication.
	Z‐FY‐DK	2D^45^	SARS‐CoV‐2	Decreases viral replication.
Cathepsin‐B and ‐L inhibition	E‐64D^45^	2D^45^	SARS‐CoV‐2	Inhibits viral replication.
Cathepsin‐B inhibition	CA‐074	2D^45^	SARS‐CoV‐2	Does not inhibit viral replication.
Autolysosomal acidification blockage	Bafilomycin	2D^45^	SARS‐CoV‐2	Does not inhibit viral replication.
PIKfyve inhibition	Apilimod	2D^45^, ^52^	SARS‐CoV‐2	Inhibits viral replication but induces concentration‐dependent apoptosis.
		3D^52^	N.A.	Ceases spontaneous beating and calcium transients.
Inward sodium ion current inhibition	Ranolazine	2D^61^	SARS‐CoV‐2	Blocks the increase of IL‐6/TNF‐α‐dependent ROS generation.
JAK inhibition	Tofacitinib	2D^61^	SARS‐CoV2	Blocks TNF‐α and IL‐6 generation and inhibits the JAK/STAT pathway.
TBK inhibition	MRT67308	3D^63^	SARS‐CoV‐2	Does not prevent viral replication and TNF expression. Sarcomere breakdown remains prevalent.
Viral protease inhibition	Ritonavir	2D, 3D^52^	N.A.	Induces apoptosis and sarcomeric disarray. Weakens contractile functions.
	Lopinavir	2D, 3D^52^	N. A.	Induces apoptosis and sarcomeric disarray. Weakens contractile functions.
Furin inhibition	Decanoyl‐RVKR‐CMK	2D^42^, ^45^	SARS‐CoV‐2	Inhibits SARS‐CoV‐2 fusion but does not reduce infection.

Abbreviations: ACE2, angiotensin‐converting enzyme‐2; COVID‐19, Coronavirus disease 2019; TMPRSS2, transmembrane protease serine 2.

## 4 FUTURE DIRECTIONS

### 4.1 Disease and gender‐specific model for viral studies

COVID‐19 exacerbates pre‐existing cardiovascular conditions and directly harms the heart muscle, rendering patients with pre‐existing heart disease particularly vulnerable to COVID‐19‐induced heart failure. In fact, hypertension, coronary heart disease, and other cardiovascular diseases are among the most common comorbidities associated with increased risk of infection and poor outcomes.^1^, ^135^, ^136^ In anticipation of unique challenges in the post‐pandemic landscape, reflecting on heretofore advancements to chart a roadmap for future direction can offer valuable insights. Remarkably, more than 20% of the studies surveyed in this review utilized heart‐on‐a‐chip technologies in some capacity to unravel the fundamental mechanisms underlying COVID‐19‐induced failure^46^, ^63^, ^74^ or to identify promising drug candidates,^52^, ^134^, ^137^ slowly unlocking the translational potential of these innovative platforms in the clinical settings (Figure 4a). While existing studies primarily focus on the role of SARS‐CoV‐2 in “healthy” cardiac tissues, it is important to generate “unhealthy” or “pathological” cardiac tissues to comprehensively elucidate the impact of preexisting heart diseases, genetic factors, and sex‐specific contributions on disease progressions (Figure 6b). Patients with preexisting heart conditions have demonstrated increased susceptibility to COVID‐19 and an increased risk of severe outcomes. Recent advancements in cardiac tissue engineering have allowed the generation of key phenotypes associated with cardiac diseases, including fibrosis,^138^, ^139^ nongenetic cardiomyopathy,^140^ and arrhythmia,^140^ through the incorporation of specific compounds or modulation of cell composition. The widespread availability of patient‐specific human iPSC lines harboring specific mutations has further enabled the generation of cardiomyocytes exhibiting functional abnormalities.^125^, ^141^, ^142^, ^143^, ^144^ Moreover, emerging evidence highlights the influence of sex‐related disparities on cellular functions, an aspect often overlooked in disease pathology. Notably, myocarditis disproportionately affects young adult males due to elevated testosterone levels and cytokines, which can exacerbate cardiac inflammation.^145^, ^146^ Thus, the development of sex‐specific engineered cardiac tissues holds the potential for investigating the effects of SARS‐CoV‐2 and other emerging viruses in a more physiologically relevant manner. The exogenous supplementation of sex hormones may be required to generate a truly specific environment, in addition to including inflammatory cytokines to appropriately capture tissue responses. These studies would pave the way for the development of models of male–female and female–male transitions. By incorporating genetically engineered cardiac tissue in the preexisting heart‐on‐a‐chip systems, a comprehensive understanding of the complex interplay between SARS‐CoV‐2 infection and sex‐specific environment can be achieved, ultimately improving the management and treatment of COVID‐19 in patients with diverse backgrounds.

To further enhance the rapid analysis of cardiac tissue function, efforts are underway to incorporate cardiac tissues into conventional 24‐ or 96‐well platforms and online sensors.^105^, ^147^, ^148^ Combination of high‐throughput drug screening capacities with advanced computational models will greatly accelerate the discovery of new drugs (Figure 6b). With the convergence of artificial intelligence and machine learning, the drug screening process is gradually shifting from trial‐and‐error to more systemic strategies. Notably, the major roadblock for the development of new drugs is the availability of sufficiently large training datasets. The development of high‐fidelity models that generate accurate and reliable data for machine learning will enable the exploration of vast drug libraries and greatly accelerate the identification of potential treatments. (Machine learning human pluripotent stem‐cell‐derived engineered cardiac tissue contractility for automated drug classification).

### 4.2 Development of atrial‐specific cardiac tissues for viral studies and drug development

The previously mentioned studies only focus on ventricular dysfunctions, while viral impacts on the atria and atrial cardiotoxicity are frequently overlooked. According to a single‐cell sequencing study reported by Litviňuková et al., atria‐specific cells, including cardiomyocytes, fibroblasts, and endothelial cells, are distinctly different than their ventricular counterparts.^149^ Atrial cardiomyocytes generate unique AP profiles, with triangle‐like AP shapes, less prominent Ito notch, shorter AP durations, and atria‐specific ion channel expression, such as IKur, IKach, T type Ca^2+^ channel, and Ca^2+^‐activated K+ channels.^111^ Atrial cardiomyocytes also express atria‐specific structural proteins, such as MLC2a, and Cx40, instead of MLC2v and Cx43 in ventricular chambers.^150^ These differences contribute to the faster but weaker atrial versus ventricular contractions, as well as the chamber‐specific disease manifestations and drug responses.^151^ Based on national‐scale cohort studies in Korea^152^ and UK,^153^ atrial fibrillation (AF), one of the most common causes of mortality in the elderly, is significantly associated with increased risk for severe complications and mortality after COVID diagnosis. New‐onset AF was considered an indicator of the adverse clinical parameters of COVID‐19, rather than an independent determinant of mortality.^154^ Thus, there is an imminent need for atria‐specific in vitro models to explore the underlying mechanisms of and potential therapeutic strategies for AF. Recently, atrial heart‐on‐a‐chip models were developed to demonstrate atrial AP profiles, forces of contraction, structural proteins, and atria‐specific sensitivity to carbochol and serotonin using iPCS‐derived atrial cardiomyocytes.^111^, ^155^, ^156^ These chamber‐specific tissue models simulate the physiological hallmarks of atrial tissues and can therefore be used to screen chamber‐specific cardiotoxicities. However, the drawbacks of these models were obviously being oversimplified with only two cell types, namely cardiomyocytes and cardiac fibroblasts. Although these two cell types primarily contribute to heart contractions and cardiac tissue formations, the lack of the endothelial network limits the sizes of developed functional tissues. The 3D atrial in vitro models were typically small, in the range of millimeters, thus, unable to create the electrical re‐entry events, aka “rotors” within the tissue geometry. The rotors are considered as a region of spiral electrical propagation that the conduction velocity of the center approximates zero. The rotors have been demonstrated with immortalized cell lines and 2D cultures, however, they are not the true recapitulation of in vivo microenvironment. Despite the limitations, the progress shed light on developing more robust preclinical testing platforms that can provide more reliable, clinically relevant information.

### 4.3 Toward vascularized cardiac and body‐on‐a‐chip models

The heart is one of the most vascularized organs in the body, with an equivalent number of cardiomyocytes and endothelial cells present in the myocardium.^57^ Its complex vasculature both maintains heart homeostasis and promotes cardiac maturation under healthy conditions.^157^ Moreover, endothelial cells serve as a physical and chemical barrier in the heart, indicating that direct administration of viruses or drugs to the engineered heart tissue may lead to an overestimation of their effects. In parallel, the immunomodulatory effects of endothelial cells and immune cells have been implicated in many heart dysfunctions. In the event of a SARS‐CoV‐2 infection, both innate and adaptive immune responses instigate a systemic surge of chemokines and cytokines. Even though SARS‐CoV‐2 infection does not productively infect endothelial cells,^62^ the prolonged inflammatory environment activates endothelial cells, thereby increasing the expression of adhesion molecules such as P‐selectin, E‐selectin, and intracellular adhesion molecule‐1, along with their respective soluble forms.^35^ This leads to the recruitment, attachment, and transmigration of immune cells into the myocardium, increasing the risk of myocarditis. Concurrently, endothelial dysfunction and inflammation catalyze the release of von Willebrand factor, which contributes to platelet adhesion and subsequent thrombus formation.^158^ These findings underscore the importance of incorporating all relevant cell types to facilitate critical crosstalk within the myocardium. Several studies have elegantly applied microfabrication and 3D printing techniques to create macrovasculature‐like open vessel lumens surrounded by cardiomyocytes.^123^, ^127^, ^129^, ^130^ A vasculogenesis‐promoting approach involving the coculture of endothelial cells, stromal cells, and cardiomyocytes has also been explored to create branching networks that exhibit capillary‐like microvasculature.^128^, ^159^, ^160^ The establishment of tissue vascularization will more accurately mimic the physiological environment of the heart and enable the study of more complex tissue‐tissue interactions, such as myocarditis and thrombosis^161^, ^162^ (Figure 6b).

We also speculate that heart‐on‐a‐chip platforms with circulating immune cells may significantly influence the trajectory of vaccine research and development, particularly in light of the observed increased incidence of myocarditis following mRNA‐based COVID‐19 vaccines across various age and sex demographics.^163^, ^164^ Although these vaccines do not directly interact with cardiovascular systems, both innate and adaptive immune responses instigated by mRNA vaccination could lead to systemic inflammation throughout the body. In rare instances, such inflammation may affect cardiac tissues, thereby causing a short episode of acute myocarditis. With this regard, heart‐on‐a‐chip platforms with immune components offer the potential for assessing immunogenicity, toxicity, and efficacy of vaccines, thereby streamlining the preclinical evaluations of vaccines prior to testing on humans.

Recent studies highlighted the significance of connecting multiple organs through a single vascular network to better understand inter‐organ biological events and improve drug screening.^108^, ^165^, ^166^, ^167^ We believe that the development of duo‐organ systems, such as lung‐heart and liver‐heart systems, will enable assessment of the direct and indirect coupling of these organs, provide insights into the impact of pulmonary inflammation on the cardiovascular system in the context of viral infection, and facilitate exploration of drug metabolism by the liver and subsequent cardiotoxicity. While the current methods of performing cell coculture and growing duo‐tissues rely on the use of media prepared by proportionally combining cell‐type‐specific culture media based on respective cell quantities, the functional phenotype of each distinct population may not be preserved. By introducing perfusable vasculature in the system, we can address media requirements by providing each tissue with its own specialized media in each compartment and connecting tissues by vascular flow. We further envision that the “body‐on‐a‐chip” systems, compromising three or more bioengineered tissues integrated into a single device, represents an emerging frontier in the quest to fully elucidate the intricate organ‐tissue interplay within the body. When Shuler and colleagues initially unveiled the body‐on‐a‐chip that can capture system interplay among the liver, kidney, and gastrointestinal tract, it sparked the creation of more complex models. Subsequently, systems featuring interconnected liver, heart, bone marrow, and skin,^165^ as well as configurations comprising gut, liver, and kidney,^168^ all interconnected through a unifying vasculature, were engineered. These advanced in vitro platforms hold the potential to faithfully recapitulate the pharmacokinetic and pharmacodynamic properties of therapeutic agents,^165^, ^166^ and to enrich understanding of viral pathogenesis within a biological milieu akin to in vivo conditions, all the while circumventing the necessity for animal models.

## 5 CONCLUSIONS

Since the development of the first 3D cardiac tissues over 20 years ago, significant advancements have been made and the field has reached a stage of maturity. Amidst the COVID‐19 pandemic, these 3D systems have proved valuable in providing insights into the direct and indirect mechanisms of virus interactions with the heart, as well as in expediting the assessment of the cardiac safety of potential therapeutics. As we reflect on these advancements, there is a sense of optimism that engineered cardiac tissues will eventually replace conventional 2D approaches in disease modeling and drug discovery. Looking ahead, we anticipate significant progress will be made in cardiac tissue engineering, including the integration of all relevant cell types (endothelial cells, resident macrophages, and circulating immune cells) in a sex and patient‐specific manner, robust maturation protocols, and high‐throughput platforms, that will collectively pave the way toward more advanced and superior cardiac tissue models.

## AUTHOR CONTRIBUTIONS

**Rick Xing Ze Lu:** Conceptualization (lead); visualization (lead); writing – original draft (lead); writing – review and editing (lead). **Yimu Zhao:** Writing – original draft (supporting); writing – review and editing (supporting). **Milica Radisic:** Conceptualization (supporting); writing – review and editing (supporting).

## CONFLICT OF INTEREST STATEMENT

M.R. and Y.Z. are inventors on a patent describing Biowire II heart‐on‐a‐chip technology that is licensed to Valo Health. They receive royalty payments. The remaining author declares no conflicts of interest.

### PEER REVIEW

The peer review history for this article is available at https://www.webofscience.com/api/gateway/wos/peer-review/10.1002/btm2.10581.

## Data Availability

The additional information in this manuscript is available from the corresponding author upon request.
